# Acrodermatitis dysmetabolica with concomitant acquired acrodermatitis enteropathica in a patient with maple syrup urine disease

**DOI:** 10.1016/j.jdcr.2023.12.009

**Published:** 2024-01-01

**Authors:** Luis E. Santaliz-Ruiz, Angélica C. Marrero-Pérez, Julio Sánchez-Pont, Oscar Nevárez-Pomales

**Affiliations:** a Department of Dermatology, University of Puerto Rico School of Medicine, San Juan, Puerto Rico; b Department of Medicine, University of Medicine and Health Sciences, Basseterre, Saint Kitts and Nevis

**Keywords:** acrodermatitis dysmetabolica, acrodermatitis enteropathica, maple syrup urine disease, skin manifestations in nutritional deficiencies, zinc deficiency

## Introduction

Zinc is crucial for the growth and differentiation of epidermal keratinocytes. Zinc deficiency may be acquired or inherited, known as acrodermatitis enteropathica (AE).^1^ Individuals with malabsorption syndromes, total parenteral nutrition, and poor zinc intake can develop acquired AE.^1^^,^^2^ Characteristic manifestations of AE include alopecia, diarrhea, and periorificial anogenital and acral dermatitis (AE-like triad).^2^ Individuals with metabolic disorders, such as maple syrup urine disease (MSUD), can exhibit an AE-like triad of entero-cutaneous manifestations known as acrodermatitis dysmetabolica (AD). Reported AD cases in MSUD have low isoleucine and normal serum zinc levels. Here, we report a case of a patient with MSUD who exhibited an AE-like triad in the presence of low levels of leucine, valine, and zinc.

## Case presentation

**Fig 1:**
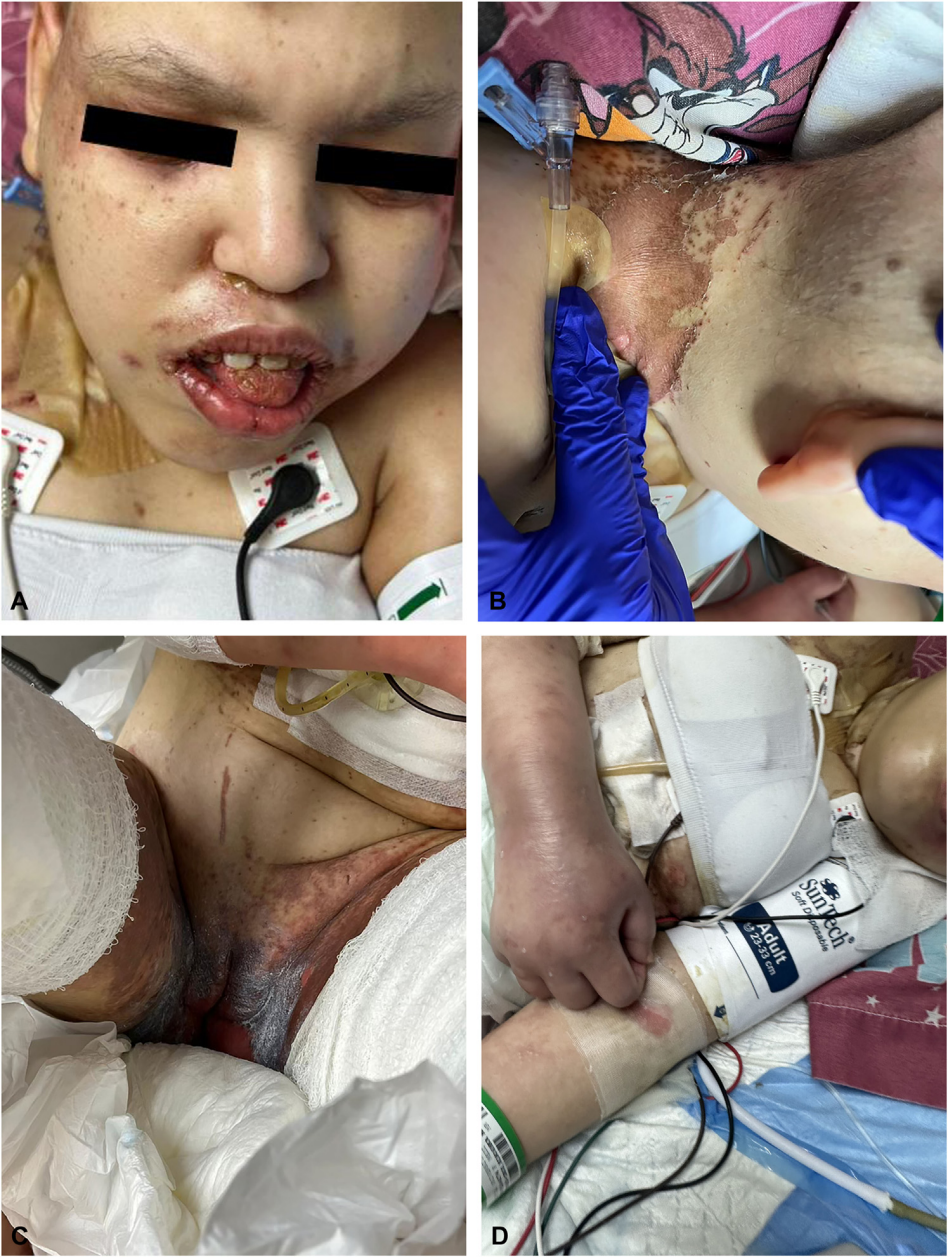
Acrodermatitis dysmetabolica and acquired acrodermatitis enteropathica lesions. Erythematous brownish patch with peripheral scale and thin-crusted plaques on **(A)** the peri-nasal, peri-ocular, and peri-oral regions, **(B)** neck, and **(C)** peri-genital regions. Erythematous-violaceous plaque with vesicles and prominent edema is present on **(D)** the dorsal aspect of the arm.

**Fig 2:**
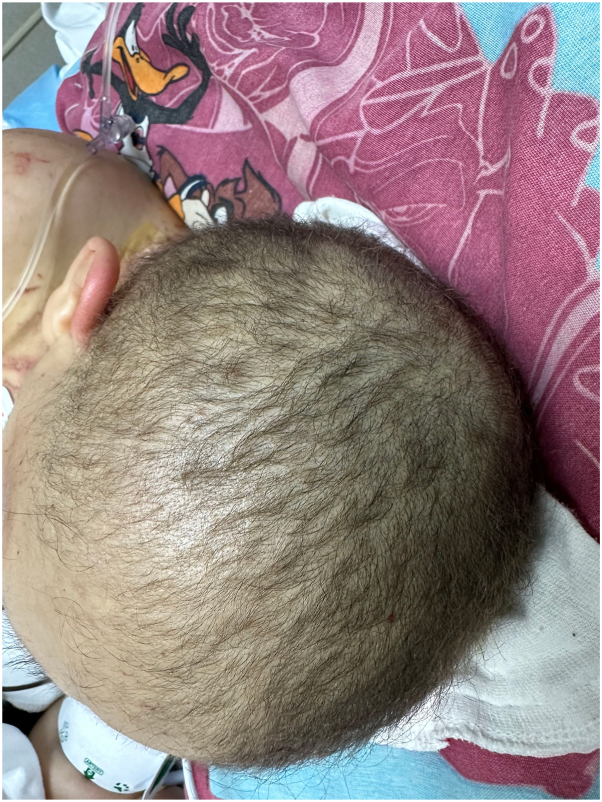
Alopecia secondary to acrodermatitis dysmetabolica and acquired acrodermatitis enteropathica. Noncicatricial hair loss with reduced hair density through scalp without perifollicular erythema.

**Fig 3:**
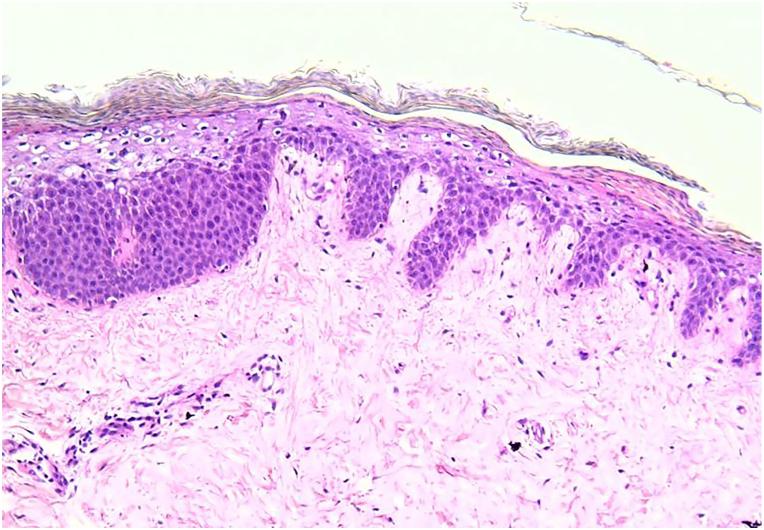
Histopathologic findings in acrodermatitis dysmetabolica and acquired acrodermatitis enteropathica. Hematoxylin-eosin–stained section of skin biopsy taken from the scaly plaques on the neck shows psoriasiform epidermal hyperplasia with the classical finding of keratinocyte pallor in the upper epidermal layer. (Hematoxylin-eosin stain; original magnification: ×10).

A 24-year-old woman with a history of MSUD since infancy presented to the emergency department with a nonpruritic cutaneous eruption, diarrhea, and hair loss for the past 2 weeks. She also had poor feeding over the same period. The skin lesions began around the eyes, nose, and mouth and spread gradually to the neck, axillae, and anogenital regions. Upon further questioning, the mother reported 1 week before the onset of rash, her daughter’s primary care physician prescribed a 7-day course of oral cephalexin for a small abscess on the axilla, which resolved after 2 days of antibiotic. Fever, chills, cough, cold symptoms, similar cutaneous manifestations in the past, recent travels, or ill contacts were denied. Medical history included gastrostomy tube and a branched-chain amino acid (BCAA)-restricted diet. The internal medicine team admitted the patient, and our dermatology service was consulted to evaluate the cutaneous eruption. Dermatologic examination found scaly erythematous-brown patches and thin-crusted plaques on the peri-oral, peri-nasal, peri-ocular regions, and neck. Similar lesions with an erythematous-violaceous base on peri-genital regions, bilateral axilla, and popliteal fossae were observed. There was prominent edema and erythema of bilateral dorsal aspect of the hands and feet (Fig 1). Diffuse nonscarring alopecia of the scalp without follicular erythema was also noted (Fig 2). There was no nail dystrophy present. Initial laboratories showed low levels of leucine (24 nmol/mL; reference: 66-183 nmol/mL), valine (32 nmol/mL; reference: 136-309 nmol/mL), and hypoalbuminemia (1.8 g/dL; reference: 3.4-5.4 g/dL). Isoleucine levels were normal (39 nmol/mL; reference: 36-107 nmol/mL), and as typically seen in MSUD, allo-isoleucine levels were increased (37 nmol/mL; reference: <5). Given the above findings, we suspected AD because of a potential deficiency of BCAAs in the MSUD diet, acquired AE, or pellagra. The patient received leucine and valine supplementations. Skin biopsy showed confluent parakeratosis and psoriasiform epidermal hyperplasia with pallor of keratinocytes in the upper epidermis, papillary edema with focal vacuolar interface changes, and a sparse lymphocytic infiltrate (Fig 3). Additional laboratories revealed normal serum niacin levels (5.3 μg/mL; reference: 0.5-8.45 μg/mL), whereas zinc levels were low (39 μg/dL; reference: 60-130 μg/dL). After clinicopathologic correlation, the patient was diagnosed with combined AD with acquired AE secondary to nutritional deficiencies of valine, leucine, zinc, and albumin. Thus, zinc and albumin were supplemented. Unfortunately, the patient succumbed to multiresistant Acinetobacter baumannii sepsis, secondary to catheter-associated bacteremia.

## Discussion

MSUD is a rare autosomal recessive disorder characterized by a deficiency of branched-chain α-ketoacid dehydrogenase, an enzyme required for degradation of BCAAs: isoleucine, leucine, and valine. Lack of branched-chain α-ketoacid dehydrogenase leads to their accumulation, causing sweet-smelling urine and sweat and neurologic sequelae. Primary management of MSUD involves dietary restriction of BCAAs while ensuring a healthy balance and catabolism avoidance.^3^ AD occurs when BCAAs reach critical low levels in MSUD diet. Characteristic clinical manifestations are known as AE-like triad.^2^^,^^3^ Although the pathogenesis of AE is known, the cause of AD remains unknown. Some studies suggest that BCAAs may play a similar role to zinc in the skin’s epidermal proliferation and keratinocyte differentiation.^2^, ^3^, ^4^ However, research on this topic is scarce. Zinc is crucial in maintaining skin and hair integrity. Its deficiency results in abnormal-appearing skin and hair keratinization, yielding an AE-like triad. Telogen effluvium is associated with zinc deficiency.^1^^,^^5^ Our patient’s hair findings were consistent with this type of nonscarring alopecia. Acquired AE may arise from poor intake of zinc and malabsorption, whereas inherited AE is caused by a mutation in the SLC39A4 gene leading to zinc malabsorption.^4^^,^^6^ AD and AE share the histopathologic feature of keratinocyte pallor in the upper epidermis.^4^^,^^7^^,^^8^ Although biopsy can be useful, it is nonspecific and can be seen with other nutritional deficiencies, such as necrolytic migratory erythema and pellagra. Therefore, diagnosis heavily relies on clinical history, physical examination, and laboratory studies.^5^^,^^7^^,^^9^

According to the medical literature, AD in MSUD resolves by supplementing deficient amino acids.^2^^,^^3^ Similarly, in typical AE, by zinc supplementation.^5^, ^6^, ^7^^,^^9^ To our knowledge, no cases of MSUD with combined AD and acquired AE have been reported to guide management. However, Kim et al^10^^,^^11^ reported an infant on a protein-restricted diet with an AE-like reaction with coexisting hypozincemia and amino acid imbalance. Lesions worsened with zinc supplementation alone but resolved when both zinc and amino acids were given.^10^^,^^11^ This supports our conjecture that AD and acquired AE may coexist in MSUD after a BCAA-restricted diet.

Our patient’s poor feeding and MSUD diet resulted in combined leucine, valine, zinc, and albumin deficiency. The absorption of zinc depends on amino acids and albumin.^3^, ^4^, ^5^, ^6^ In MSUD, hypoalbuminemia elicits albumin unbound zinc to bind BCAAs, which in turn increases their urinary excretion and further contributes to the cooccurrence of AD and acquired AE. Despite appropriate supplementations of leucine, valine, zinc, and albumin, regretfully, the patient passed before any clinical improvements could be ascertained.

In conclusion, a high index of suspicion for concomitant AD and acquired AE must be entertained in any patient with MSUD with an AE-like triad. BCAA-restricted diet is key in managing MSUD; however, a fine balance must be exercised to avoid surplus and neurologic sequelae vs critically low levels of BCAAs that may trigger AD. Monitoring serum BCAAs, zinc, niacin, and albumin levels in MSUD is paramount to treat accordingly.

## Conflicts of interest

None disclosed.
